# Supplementary material for: Excitonic Bloch–Siegert shift in CsPbI3 perovskite quantum dots
Source: Nat Commun. 2022 Sep 22;13:5559. doi: 10.1038/s41467-022-33314-9 (PMC9500032; doi:10.1038/s41467-022-33314-9)
Supplement: Supplementary file 1 — Supplementary Information [file 41467_2022_33314_MOESM1_ESM.pdf]

Supplementary Information for:

Excitonic Bloch-Siegert shift in $CsPbI_3$ perovskite quantum dots

Li *et al.*

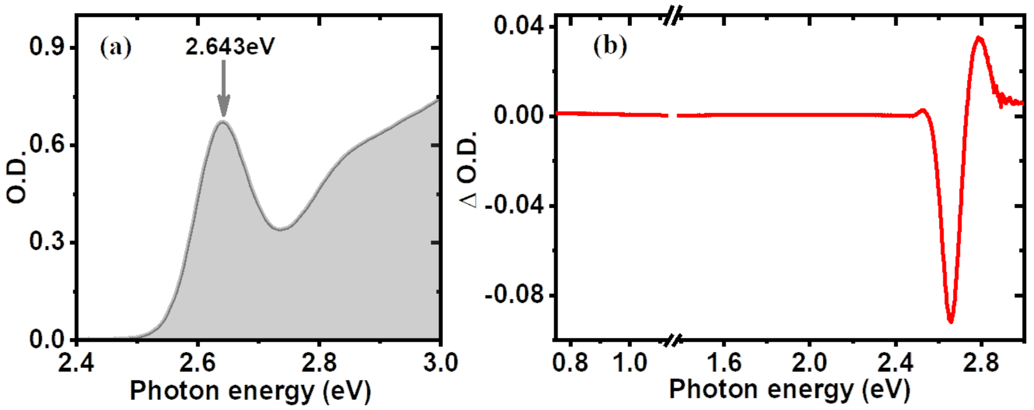

Supplementary Figure 1. Spectra of $CsPbBr_3$ QDs. (a) Steady state absorption spectrum and (b) transient absorption spectrum at 2 ps of the 3.9 nm $CsPbBr_3$ QDs. Pump photon energy is at around 3.02 eV (410 nm) and is linearly polarized.

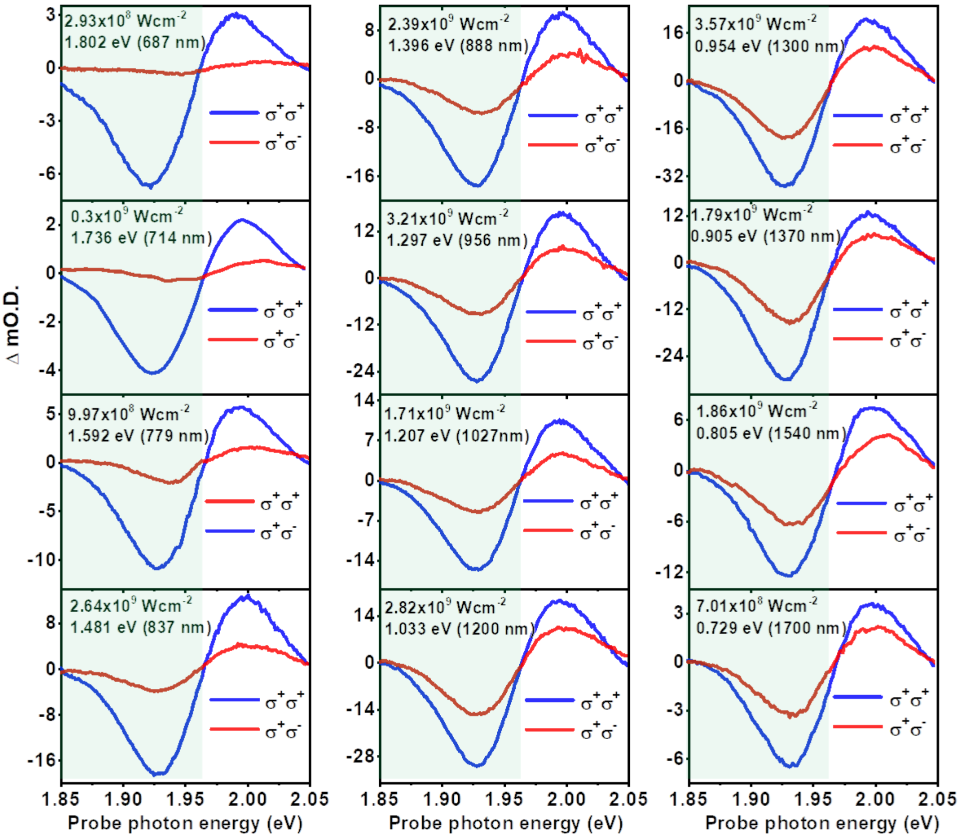

Supplementary Figure 2. TA spectra. Pump photon energy dependence of the nominal Stark ($\sigma^+\sigma^+$) and Bolch-Siegert ($\sigma^+\sigma^-$) shift TA spectra observed in $CsPbI_3$ quantum dots. The pump photon energies and intensities are indicated in each panel, and the shallow green shaded regions are used for the spectral- weight-transfer calculations for obtain the ratio of $\sigma^+\sigma^-/\sigma^+\sigma^+$ in the main manuscript.

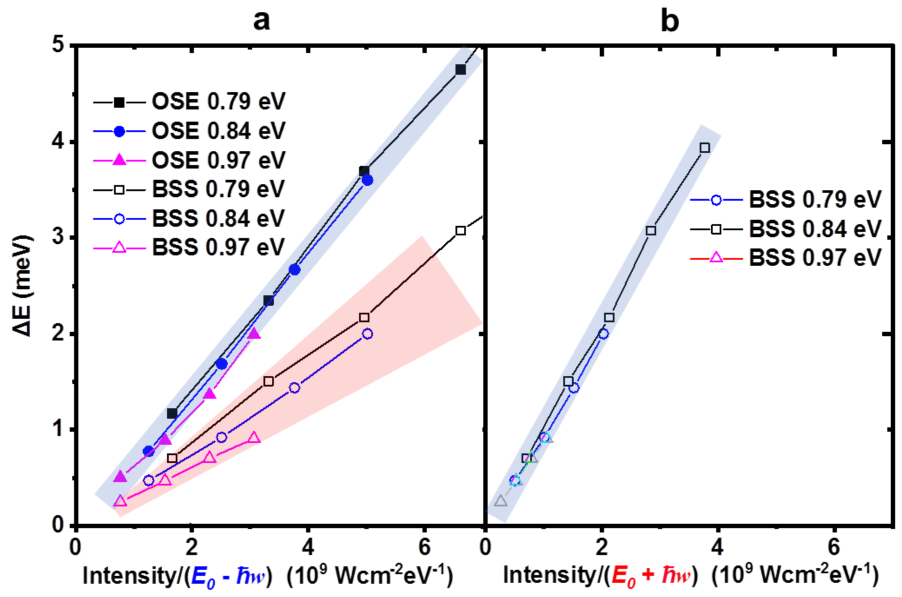

Supplementary Figure 3. Plots of $\hbar\omega_{ph}$-dependent OSE and BSS signals. (a) Plots of nominal energy shifts induced by OSE and BSS (measured with $\sigma^+\sigma^+$ and $\sigma^+\sigma^-$ configurations, respectively) as a function of pump intensity over (E_0 - $\hbar\omega_{ph}$), where E_0 is the exciton energy. Three different pump photon energies $\hbar\omega_{ph}$ are included in the plot. (b) Re-plot of BSS signals as a function of pump intensity over (E_0 + $\hbar\omega_{ph}$).

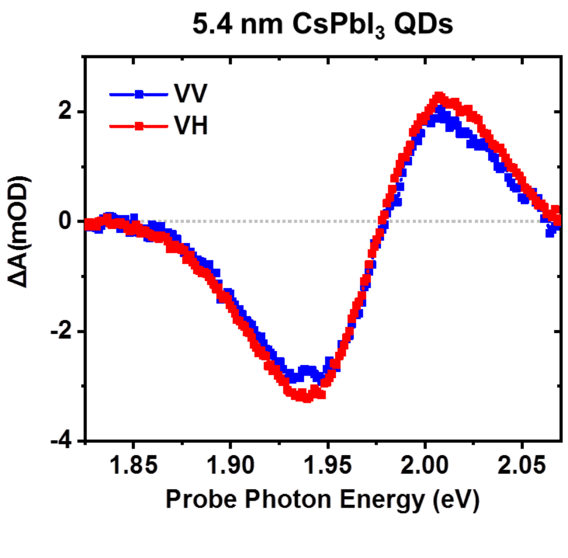

Supplementary Figure 4. Linear-polarization OSE. Linear-polarization pump-probe (VV and VH) measurements of the OSE in 5.4 nm $CsPbI_3$ QDs ($\hbar\omega_{ph}$: 1.72 eV; 0.4 GW/cm^2).

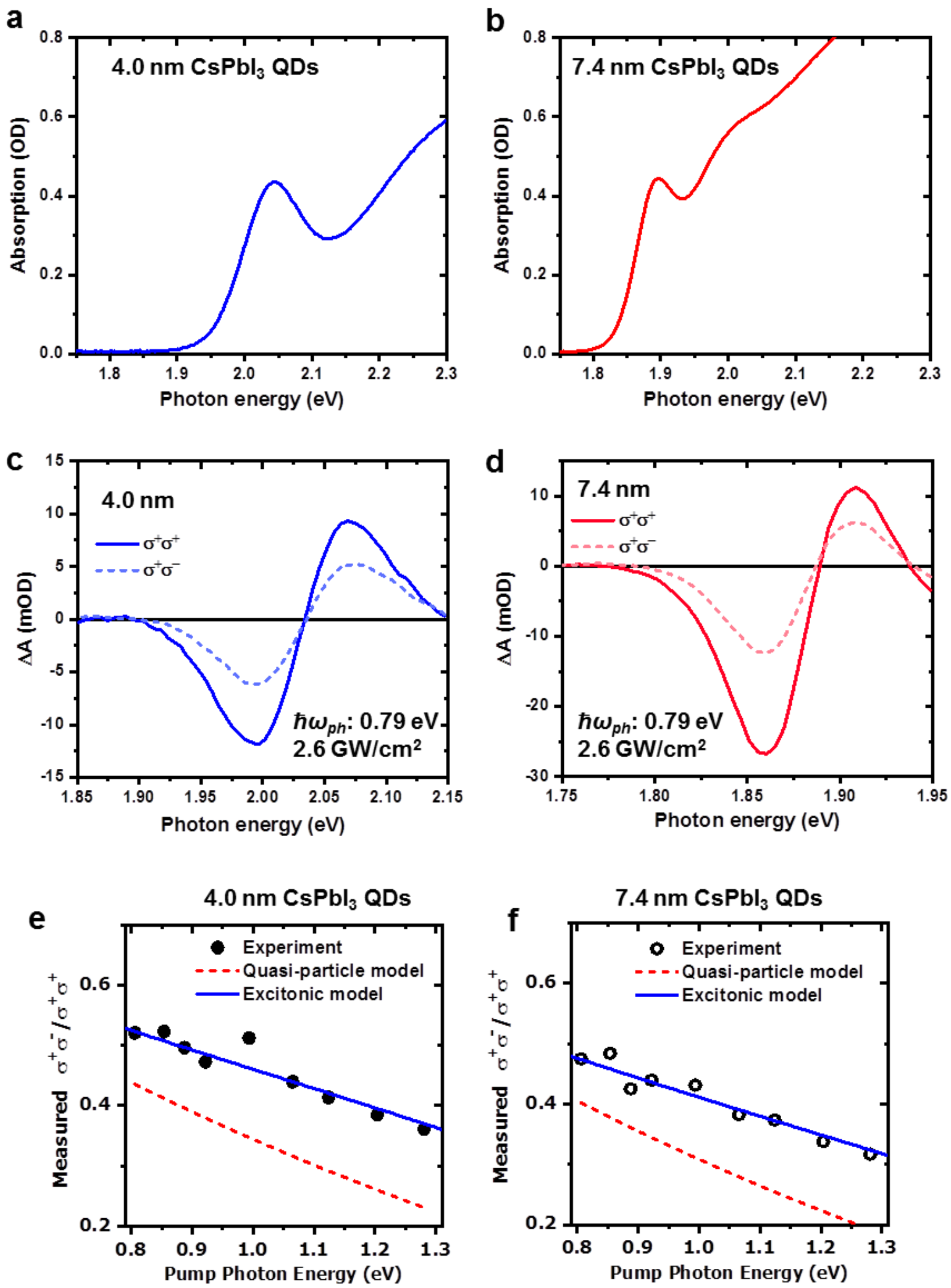

Supplementary Figure 5. QD size-dependent measurements. (a,b) Absorption spectra of another two sets of $CsPbI_3$ QDs, with their average edge lengths of (a) 4.0 nm and (b) 7.4 nm. (c,d) TA spectra measured with $\sigma^+\sigma^+$ (solid) and $\sigma^+\sigma^-$ (dashed) configurations for (c) 4.0 nm and (d) 7.4 nm QDs. The pump conditions are indicated. (e,f) Ratio between signals obtained with $\sigma^+\sigma^-$ and $\sigma^+\sigma^+$ configurations ($\sigma^+\sigma^-/\sigma^+\sigma^+$) for (e) 4.0 nm and (f) 7.4 nm QDs. The experimental data are in black circles, fittings using the quasi-particle and excitonic models are shown as red dashed and blue solid lines, respectively.

Supplementary Table 1. Summary of the μ_{01}, μ_{12} and E_{XX} parameters for QDs of three sizes.

	μ_{01} (Debye)	μ_{12} (Debye)	E_{XX} (meV)
**4.0 nm**	26	19	90
**5.4 nm**	34	28	65
**7.4 nm**	50	41	48

Supplementary Text: Optical Stark and Bloch-Siegert shift of a two-energy level system.

For a simple two-energy level system (ground state *G* and excited state *Ex*) with transition dipole moment of μ_{eg} and resonant energy of $\hbar\omega_{eg}$, we can resolve two situations of the interaction with the detuned light field: the corotating interaction component and the counterrotating interaction, corresponding to the optical Stark and Bloch-Siegert shifts, respectively. Following eq 1 in the main manuscript, for the corotating part, the Hamiltonian can be written as:

$$\hat{\mathrm{H}}_{Stark} = \hbar\omega_{eg}\hat{\mathrm{c}}^{\dagger}\hat{\mathrm{c}}^{-} + \hbar\omega_{ph}\hat{\mathrm{a}}^{\dagger}\hat{\mathrm{a}}^{-} + \hbar\lambda(\hat{\mathrm{c}}^{\dagger}\hat{\mathrm{a}}^{-} + \hat{\mathrm{c}}^{-}\hat{\mathrm{a}}^{\dagger}) \dots\dots\dots (1)$$

In the subspace containing the excited matter-state $|Ex,\ 0\rangle$ and the one-photon dressed up Floquet state $|G, +1\rangle$, (1) becomes:

$$\hat{\mathrm{H}}^{up}_{Stark} = \hbar\omega_{eg}\begin{bmatrix}1 & 0\\ 0 & 0\end{bmatrix} + \hbar\omega_{ph}\begin{bmatrix}0 & 0\\ 0 & 1\end{bmatrix} + \hbar\lambda\begin{bmatrix}0 & 1\\ 1 & 0\end{bmatrix} \dots\dots\dots (2)$$

The diagonalized eigen energy is

$$E^{up}_{Stark} = \frac{\hbar}{2}(\omega_{eg} + \omega_{ph}) \pm \frac{\hbar}{2}\sqrt{(\omega_{eg} - \omega_{ph})^2 + 4\lambda^2} \;\dots\dots (3)$$

For large negative detuning and moderate coupling strength, i.e., $\lambda \ll \omega_{eg} - \omega_{ph}$, (3) reduces to:

$$E^{up}_{Stark} = \frac{\hbar}{2}(\omega_{eg} + \omega_{ph}) \pm \frac{\hbar}{2}(\omega_{eg} - \omega_{ph}) \pm \frac{\hbar\lambda^2}{(\omega_{eg}-\omega_{ph})} \;\dots\dots (4)$$

For large detuning, only the high-energy level containing the matter property can be coupled to the probe pulse, whose energy is:

$$E_{Stark}^{up}(high) = \hbar\omega_{eg} + \frac{\hbar\lambda^2}{(\omega_{eg} - \omega_{ph})} \dots\dots\dots (5)$$

It is easy to prove that, in the subspace containing the ground matter-state $|G,\ 0\rangle$ and the one-photon dressed down Floquet state $|Ex, -1\rangle$, the low-energy eigen state carrying the matter property is:

$$E_{Stark}^{down}(low) = -\frac{\hbar\lambda^2}{(\omega_{eg} - \omega_{ph})} \dots\dots\dots (6)$$

Thus, the final optical Stark shift that can be observed from the transition between the high-energy and low-energy eigen states is:

$$\delta E_{Stark} = E_{Stark}^{up}(high) - E_{Stark}^{down}(low) - \hbar\omega_{eg} = \frac{2\hbar\lambda^2}{(\omega_{eg} - \omega_{ph})} \dots\dots (7)$$

For the counterrotating part that corresponding to the Bloch-Siegert shift, the Hamiltonian can be written as:

$$\hat{H}_{Bloch-Siegert} = \hbar\omega_{eg}\hat{c}^{\dagger}\hat{c}^{-} + \hbar\omega_{ph}\hat{a}^{\dagger}\hat{a}^{-} + \hbar\lambda(\hat{c}^{\dagger}\hat{a}^{\dagger} + \hat{c}^{-}\hat{a}^{-}) \dots\dots\dots (8).$$

In the subspace containing the excited matter-state $|Ex,\ 0\rangle$ and the one-photon dressed down Floquet state $|G, -1\rangle$, (8) becomes:

$$\hat{H}_{Bloch-Siegert}^{down} = \hbar\omega_{eg}\begin{bmatrix}1 & 0\\ 0 & 0\end{bmatrix} + \hbar\omega_{ph}\begin{bmatrix}0 & 0\\ 0 & -1\end{bmatrix} + \hbar\lambda\begin{bmatrix}0 & 1\\ 1 & 0\end{bmatrix} \dots\dots\dots (9)$$

The diagonalized eigen energy is

$$E_{Bloch-Siegert}^{down} = \frac{\hbar}{2}(\omega_{eg} - \omega_{ph}) \pm \frac{\hbar}{2}\sqrt{(\omega_{eg} + \omega_{ph})^2 + 4\lambda^2} \dots\dots (10)$$

since $\lambda \ll \omega_{eg} + \omega_{ph}$, (10) reduces to:

$$E_{Bloch-Siegert}^{down} = \frac{\hbar}{2}(\omega_{eg} - \omega_{ph}) \pm \frac{\hbar}{2}(\omega_{eg} + \omega_{ph}) \pm \frac{\hbar\lambda^2}{(\omega_{eg}+\omega_{ph})} \dots\dots (11)$$

The high-energy level carrying the matter property is:

$$E^{down}_{Bloch-Siegert}(high) = \hbar\omega_{eg} + \frac{\hbar\lambda^2}{(\omega_{eg}+\omega_{ph})} \;\ldots\ldots\ldots\ldots\ldots\ldots\ldots\ldots\ldots (12)$$

Similarly, in the subspace containing the ground matter-state $|G,\,0\rangle$ and the one-photon dressed up Floquet state$|Ex,+1\rangle$, the low-energy level carrying the matter property is:

$$E^{up}_{Bloch-Siegert}(low) = -\frac{\hbar\lambda^2}{(\omega_{eg}+\omega_{ph})} \ldots\ldots\ldots (13)$$

Thus, the observed Bloch-Siegert shift is

$$\delta E_{\text{Bloch-Siegert}} = E^{down}_{\text{Bloch-Siegert}}(high) - E^{up}_{\text{Bloch-Siegert}}(low) - \hbar\omega_{eg}$$

$$= \frac{2\hbar\lambda^2}{(\omega_{eg}+\omega_{ph})} \ldots\ldots (14)$$

The ratio of the observed Bloch-Siegert shift to that of the optical Stark shift is $\frac{\omega_{eg}-\omega_{ph}}{\omega_{eg}+\omega_{ph}}$.
